# ﻿Gems of the southern Japanese seas – four new species of *Edwardsianthus* (Anthozoa, Actiniaria, Edwardsiidae) with redescriptions of two species

**DOI:** 10.3897/zookeys.1076.69025

**Published:** 2021-12-10

**Authors:** Takato Izumi, Takuma Fujii

**Affiliations:** 1 Molecular Invertebrate Systematics and Ecology Laboratory, Department of Biology, Chemistry, and Marine Sciences, Faculty of Science, University of the Ryukyus, 1 Senbaru, Nishihara, Okinawa 903-0213, Japan University of the Ryukyus Nishihara Japan; 2 Kagoshima City Aquarium, 3-1 Honko-shinmachi, Kagoshima, 892-0814, Japan Kagoshima City Aquarium Kagoshima Japan; 3 International Center for Island Studies Amami Station, Kagoshima University,15-1 Naze-Minatomachi, Amami, Kagoshima 894-0026, Japan Kagoshima University Amami Japan; 4 The Kagoshima University Museum, 1-21-30 Korimoto, Kagoshima 890-0065, Japan The Kagoshima University Museum Korimoto Japan

**Keywords:** Cnidaria, cnidae, Kochi, Nansei Islands, nemathybomes, northernmost distribution limit, Pacific Ocean, phylogeny, taxonomy

## Abstract

*Edwardsianthus* England, 1987 is a genus of Edwardsiidae, a family of burrowing and worm-like sea anemones characterized by lacking four mesenteries in the first cycle and containing only one type of nematocysts in nemathybomes. Until now, this genus has accommodated only two species since its establishment and has been recorded only from Indo-West Pacific regions. In this study, six species are reported from Japan: two are previously known species, *E.pudicus* (Klunzinger, 1877) and *E.gilbertensis* (Carlgren, 1931); four are new species, *E.carbunculus***sp. nov.**, *E.sapphirus***sp. nov**., *E.smaragdus***sp. nov.**, and *E.amethystus***sp. nov.** Based on these results, the diagnostic features of the genus are revised.

## ﻿Introduction

The superfamily Edwardsioidea, re-established by Rodríguez et al. (2014), consists of only one family, Edwardsiidae Andres, 1881. This family is a large taxon in the order Actiniaria with ca. 95 nominal species (Gusmão et al. 2020). Most edwardsiids are burrowers in a broad range of soft substrates, such as sand or mud, others can bore into skeletons of dead coral in caves or rock crevices (Carlgren, 1892; Dnyansagar et al. 2018; Izumi and Fujita, 2018; Sanamyan et al. 2018), whereas a few species can live in ice (Daly et al. 2013) or in homoscleomorph sponges (Izumi et al. 2018). Edwardsiids are characterized by worm-like bodies, absence of basal disks, and eight perfect mesenteries in the first cycle, unlike almost all other sea anemones, which have 12 perfect mesenteries, excluding a few exceptional taxa (e.g., HalcampulactidaeGusmão et al. 2019). This simplified mesenterial arrangement of edwardsiids is similar to that of “Edwardsia-stage” larvae (Duerden, 1899) of several actiniarian species that have 12 or more mesenteries as adults (Uchida and Soyama, 2001). As a result, worm-like edwardsiids had been hypothesized to be the common ancestral form of actiniarians (McMurrich, 1891; Hyman, 1940). However, several studies have asserted that Edwardsiidae is a derived lineage and that the simplified mesenterial arrangement of this family is a derived character (Manuel, 1981; Daly, 2002). Recent phylogenetic studies by Rodríguez et al. (2014) and Gusmão et al. (2019) have reinforced the latter hypothesis.

The genus *Edwardsianthus* England, 1987 was established in order to rearrange the type genus of Edwardsiidae, *Edwardsia* de Quatrefages, 1842. England (1987) divided *Edwardsia* into three genera: *Edwardsia*, *Edwardsioides* Danielessen, 1890, and *Edwardsianthus* England, 1987. *Edwardsioides* was synonymized with *Edwardsia* by Carlgren (1921), which was revoked by England (1987). *Edwardsioides* was again synonymized with *Edwardsia* (Fautin, 2007) and thus only *Edwardsianthus and Edwardsia* remain as valid genera (Daly & Fautin, 2021). Since the establishment by England (1987), no additional species of *Edwardsianthus* have been discovered, and currently this genus still contains only two species (Daly & Fautin, 2021): *E.pudicus* (Klunzinger, 1877) and *E.gilbertensis* (Carlgren, 1931). Consequently, the genus *Edwardsia* remains the largest genus in the family with more than 60 species (Fautin, 2016; Daly & Fautin, 2021).

*Edwardsianthus* specimens have been collected from a broad range in the Indo-West Pacific region (Fautin, 2013). However, until now there has been only one record of an *Edwardsianthus* species from Japanese waters; *E.gilbertensis* from Ishigaki Island, Okinawa (Uchida & Soyama, 2001). This reference also mentioned Edwardsianthuscf.pudica from Onagawa, Miyagi as reported in Uchida (1941), but this observation has not been confirmed (Yanagi, 2006).

During recent surveys of Japanese waters, we recorded two previously described species of *Edwardsianthus* and also collected specimens of four undescribed species. These undescribed species have tentacles in more vivid colors than the two other ones, and they share the same particular morphological genus characters. Moreover, in our phylogenetic analysis, these undescribed species were found within the clade of *Edwardsianthus* and monophyletic with the other two species.

We formally describe the new species as *Edwardsianthuscarbunculus* sp. nov., *E.smaragdus* sp. nov., *E.sapphirus* sp. nov., and *E.amethystus* sp. nov., and redescribe the two existing species, *E.pudicus* and *E.gilbertensis*. Furthermore, we revise the definition of *Edwardsianthus* to accommodate the four new species. Since neither the family, the genus, nor its species had names in the Japanese language, we designate Japanese names to all these taxa.

## ﻿Materials and methods

### ﻿Specimen collection and preservation

**Figure 1.**
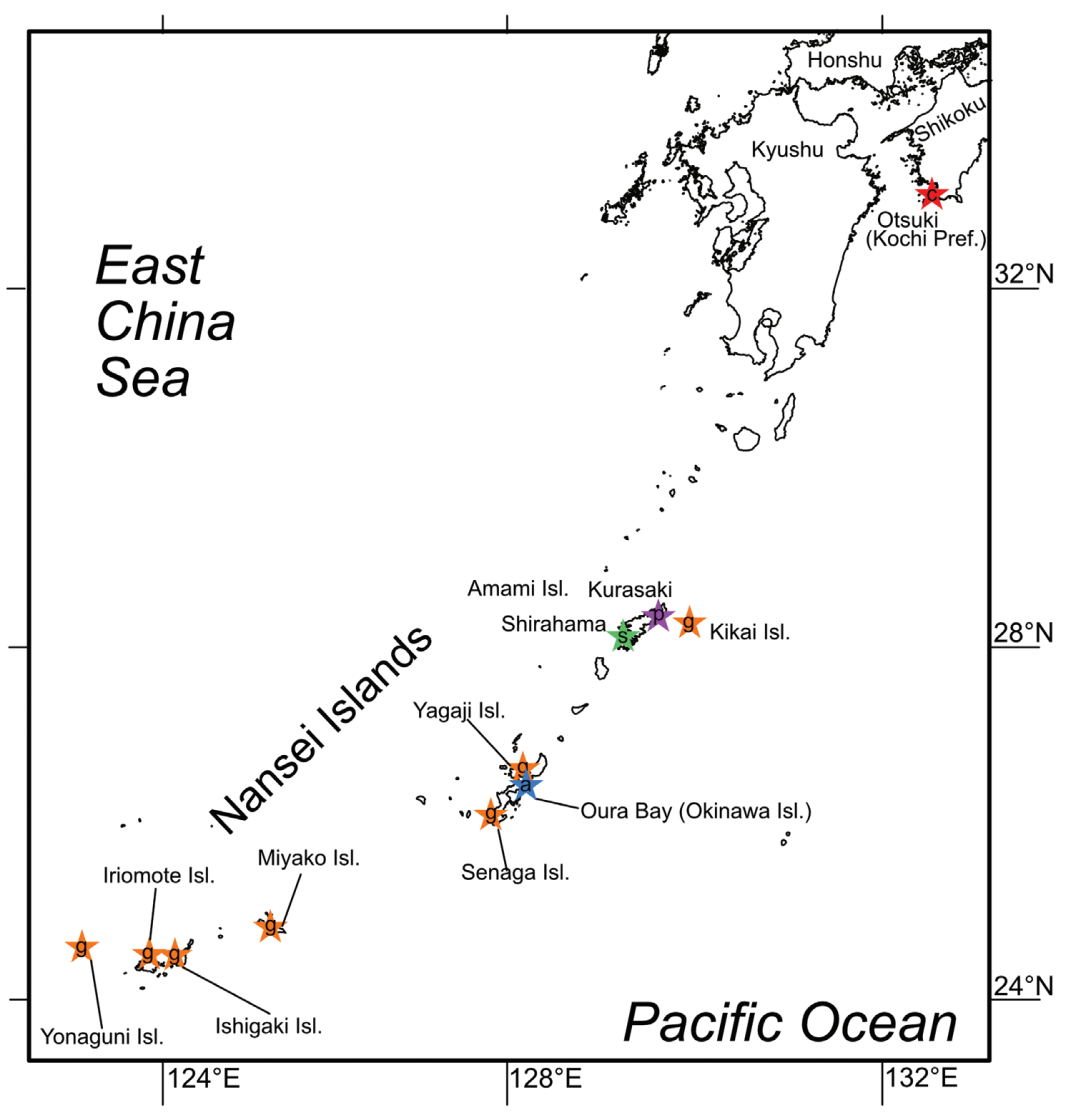
Localities of the specimens of the *Edwardsianthus* species collected in this study. Purple star marked by p indicates collection locality of *Edwardsianthuspudicus*; orange stars are those of *E.gilbertensis*; red star marked by c is *E.carbunculus* sp. nov.; blue star marked by a is *E.sapphirus* sp. nov.; green star marked by s is *E.smaragdus* sp. nov.

Specimens of *Edwardsianthus* used in the present study were collected from southern Japan (Fig. 1). Specimens of *E.gilbertensis* were collected by digging in shallow, submerged areas at low tides, whereas the other species were sampled during scuba diving. They were dug out by using a shovel and a sieve, or by hand. The specimens were generally kept undisturbed in aquaria for several hours to several days after collection, as long possible, until they were completely relaxed and had their tentacles extended. Then, specimens were anesthetized with magnesium chloride solution, magnesium sulfate solution or l-menthol, and finally fixed in 5–10% (v/v) seawater formalin solution. The examined specimens were eventually deposited in the National Museum of Nature and Science, Tokyo (**NSMT**) or in the Coastal Branch of the Natural History Museum and Institute, Chiba (**CMNH**).

### ﻿Preparation of histological sections

Histological sections of all specimens were made following standard protocols (Presnell and Schreibman, 1997); The materials were dissected by scissors, serially dehydrated by ethanol and xylene, embedded in paraffin, and sliced into serial sections each 8–10 μm thick. Thereafter, sections were mounted on glass slides. All sections were stained by hematoxylin and eosin, and finally they were mounted using the medium EUKITT (ORSAtec).

### ﻿Observation of cnidae

Cnidae were extracted from the tentacles, actinopharynx, nemathybomes, column, and filaments. Concerning the column of some *Edwardsianthus* species, no or few cnidocytes were observed, and the few observed capsules were broken or almost of the same type as those observed in their nemathybomes. In those cases, we did not describe the cnidom of column because it is possible that these cnidae were contaminants from broken nemathybomes. Images of the cnidae were obtained by differential interference contrast microscopy following the method of Yanagi et al. (2015). For each capsule, length and width were measured from the images using ImageJ v. 1.49 (Rasband, 1997–2012). Size distributions were processed and values of means and standard deviations were calculated in Microsoft Excel 2013. The nomenclature of cnidae followed Mariscal (1974). Thus, although England (1987) designated the large basitrichs in nemathybomes as “pterotrichs” and “microbasic t-mastigophores”, we unified the name of such cnidocysts as “basitrichs” following the nomenclature of Mariscal (1974).

### ﻿PCR and DNA sequencing

DNA was extracted from subsamples of each tissue that were preserved in 99% ethanol by using ChargeSwitch gDNA Micro Tissue Kit (Invitrogen). In addition, some tissue samples for DNA were processed following the guanidine extraction protocol (Sinniger et al. 2010). PCR amplifications were performed in 10 µL (or 25 µL) reaction volume, consisting of 0.4 (1.0) µL of 25 µM forward and reverse primers, 2.0 (5.0) µL of EmeraldAmp PCR Master Mix (TaKaRa), and 3.4 (8.5) µL of distilled water. For PCR amplifications, two mitochondrial markers, 12S, 16S rDNA, and a nuclear marker, 18S rDNA, were amplified. The primers and amplification conditions are shown in Table 1. Amplifications were performed using traditional molecular markers of Actiniaria; mitochondrial 12S rDNA and 16S rDNA, and nuclear 18S rDNA. PCR methods and protocols followed methods of preceding phylogenetic studies (Medlin et al. 1988; Apakupakul et al. 1999; Geller and Walton, 2001; Medina et al. 2001; Sinniger et al. 2005) referring to Rodríguez et al. (2014). The PCR products were processed using exonuclease I and shrimp alkaline phosphate (ExoSAP-IT; Thermo Fisher) before sequencing. Sequencing reactions were performed using BigDye Terminator Cycle Sequencing Ready Reaction Kit v3.1 (Applied Biosystems) and using PCR primers (12S, 16S) or PCR primers and internal primers (18S; Table 1). We used four internal primers (two forward and two reverse) for 18S (Apakupakul et al. 1999). Sequencing was performed using an ABI 3500xL Genetic Analyzer (Applied Biosystems). The sequence of each marker was individually assembled using GeneStudio ver. 2.2.0.0 (http://genestudio.com).

**Table 1.** Primers and protocols of polymerase chain reactions of every molecular marker.

Marker		Primer	Sequences (5‘-3‘)	PCR protocol	Reference
12S		12S1a	TAAGTGCCAGCAGACGCGGT	(95 °C for 4 min) + 4 × [(94°C for 30 sec) → (50°C for 1 min) → (72°C for 2 min)]+30 × [(94°C for 30 sec) → (55°C for 1 min) → (72°C for 2 min)] + (72°C for 4 min)	Sinniger et al. (2005)
		12S3r	ACGGGCAATTTGTACTAACA		
16S		ANEM16SA	CACTGACCGTGATAATGTAGCGT	(95°C for 4 min) + 30 × [(95°C for 30 sec) → (46°C for 45 sec) → (72°C for 1 min)] + (72°C for 5 min)	Geller and Walton (2001)
		ANEM16SB	CCCCATGGTAGCTTTTATTCG		
		16Sant1a	GCCATGAGTATAGACGCACA	(95°C for 4 min) + 30 × [(95°C for 30 sec) → (46°C for 45 sec) → (72°C for 1 min)] + (72°C for 5 min)	Sinniger et al. (2005)
		16SbmoH	CGAACAGCCAACCCTTGG		
18S	PCR	18SA	AACCTGGTTGATCCTGCCAGT	(94°C for 4 min) + 35 × [(94°C for 20 sec) → (57°C for 20 sec) → (72°C for 1 min 45 sec)] + (72°C for 7 min)	Medlin et al. (1988) Apakupakul et al. (1999)
		18SB	TGATCCTTCCGCAGGTTCACCT		
	Only sequence	18SL	CCAACTACGAGCTTTTTAACTG		
		18SC	CGGTAATTCCAGCTCCAATAG		
		18SY	CAGACAAATCGCTCCACCAAC		
		18SO	AAGGGCACCACCAGGAGTGGAG		

### ﻿Phylogenetic analyses

The phylogenetic analyses were performed on the family Edwardsiidae. The base sequences used in phylogenetic analyses are shown in Table 2. Each dataset was aligned using MAFFT ver. 7.402 (Katoh and Standley, 2013) under the default settings. Ambiguously aligned regions were eliminated using Gblocks ver. 0.91b (Castresana, 2002) with the type of DNA sequences and in default parameters except allowing small final blocks and gap positions within the final blocks. The obtained data were processed using Kakusan 4 (Tanabe, 2011) to select the appropriate substitution models for the RAxML and MrBayes analyses (Table 3). In the concatenated dataset, substitution parameters were estimated separately for each gene partition. The maximum-likelihood (ML) analysis was performed using RAxML-VI-HPC (Stamatakis, 2006), with substitution models recommended by Kakusan 4 and evaluated using 100 bootstrap replicates. Bayesian inference (BI) was conducted using MrBayes ver. 3.2.6 (Ronquist and Huelsenbeck, 2003) with substitution models recommended by Kakusan 4. Two independent runs of the Markov Chain Monte Carlo were performed simultaneously for 5×10^6^ generations; trees were sampled every 100 generations, and the average standard deviation of split frequencies (ASDSF) every 100,000 generations were calculated. As the ASDSF was calculated on the basis of the last 75% of the samples, the initial 25% of the sampled trees were discarded as burn-in.

**Table 2.** Base sequences in the phylogenetic analyses. Sequences indicated by accession numbers were obtained from GenBank, and those indicated by bold were newly obtained in this study. *Synactinernuschuraumi* (Actinernidae) were for the outgroups of phylogenetic analysis of Edwardsiidae.

Higher taxon			Family	Genus	Species	Localities	Catalog numbers	12S	16S	18S
Actiniaria	Anenthemonae	Edwardsioidea	Edwards	*Edwardsianthus*	*pudicus*	Amami Island	NSMT-Co 1702	**LC649467**	**LC649475**	**LC649483**
					*gilbertensis*	Ishigaki Island	CMNH-ZG 6527	–	**LC649481**	**LC649489**
					*gilbertensis*	Kataburu_Yonaguni	NSMT-Co 1701	**LC649468**	**LC649476**	**LC649484**
					*carbunculus*	Otsuki_Kochi	CMNH-ZG 05954	**LC649472**	**LC649480**	**LC649488**
					*sapphirus*	Oura Bay	CMNH-ZG 09761	**LC649469**	**LC649477**	**LC649485**
					*smaragdus*	Amami Island	CMNH-ZG 09762	**LC649471**	**LC649479**	**LC649487**
					*amethystus*	Oura Bay	CMNH-ZG 09763	**LC649470**	**LC649478**	**LC649486**
				*Tempuractis*	*rinkai*	Misaki	NSMT-Co 1573	**LC649473**	**LC649482**	**LC649490**
				*Edwardsia*	*japonica*			GU473274	GU473288	GU473304
					*timida*			GU473281	-	GU473315
				*Edwardsianthus*	*gilbertensis*			EU190728	EU190772	EU190859
				*Scolanthus*	*shrimp*			MN200242	MN200264	MN200245
					*celticus*			MN200251	MN200244	MN200240
				*Nematostella*	*vectensis*			EU190750	AY169370	AF254382
			Actinernidae	*Synactinernus*	*churaumi*	Off Ishigaki Island	NSMT-Co 1661	**LC649474**	LC484641	LC484636

**Table 3.** The substitution models of phylogenetic analyses on each marker.

	Mitochondrial		Nuclear
	12S rDNA	16S rDNA	18S rDNA
ML analysis	GTR+Gamma	GTR+Gamma	GTR+Gamma
Bayesian inference	HKY85+Gamma	HKY85+Gamma	K80+Gamma

All constructed Maximum Likelihood and Bayesian trees were rooted and combined using FigTree ver. 1.4.3 (http://tree.bio.ed.ac.uk/software/figtree/).

## ﻿Results and discussion

### Order Actiniaria Hertwig, 1882

**Suborder Anenthemonae Rodriguez & Daly, 2014**

**Superfamily Edwardsioidea Andres, 1881**

**Family Edwardsiidae Andres, 1881**

#### ﻿Genus Edwardsianthus

Taxon classificationAnimaliaActiniariaEdwardsiidae

England, 1987

5B726EEA-8CCF-56B8-B680-BA201207CCF6

##### Diagnosis

(revised from the diagnosis given by England, 1987). Body divisible into physa, scapus, and capitulum. physa short, without nemathybomes or cuticle. Scapus long, generally with nemathybomes but sometimes without, sunk in mesoglea; cuticle present. Tentacles usually 20, inequal number at inner and outer cycle: five-eight inner and 12–15 outer. Siphonoglyph weak or absent, ventral. Mesenteries eight macrocnemes and six pairs of microcnemes, minute and restricted to distal part of column. Microcnemes never paired with macrocnemes. Gametogenic tissue, filaments, and parietal and retractor muscles on macrocnemes only. Parietals well developed; retractors strong-diffuse to restricted-reniform. Cnidom: spirocysts, basitrichs, microbasic *p*-mastigophores.

##### Type species.

*Edwardsiapudica* Klunzinger, 1877 (currently recognized as *Edwardsianthuspudicus* (Klunzinger, 1877); the genus name is masculine). Type locality is Egypt, Red Sea.

##### Derivation of Japanese name.

This name is constructed from *nanyo* (south sea), *mushimodoki-ginchaku* (worm-like sea anemone).

##### Remarks.

Since England (1987) established this genus, no new species were described in addition to the two that were already known. This study revises the diagnosis of the genus for the first time in the 30 years since its original description, including new evidence for four new species.

In the present study, sea anemones resembling *Edwardsianthus* were collected from several Japanese localities (Fig. 1). According to our analyses, these edwardsiids were shown to belong to the same phylogenetic clade as *E.pudicus* and *E.gilbertensis* (Fig. 10) and also shared the same mesenterial arrangement, i.e., lacking four microcnemes on the first mesenterial cycle. Therefore, these new species fit well with the definition of *Edwardsianthus* given by England (1987). Thus, we have placed these four new species within *Edwardsianthus* as *Edwardsianthuscarbunculus* sp. nov., *E.sapphirus* sp. nov., *E.smaragdus* sp. nov., and *E.amethystus* sp. nov..

England (1987) also stated that the genus *Edwardsianthus* has only one type of basitrich in the nemathybomes. However, *Edwardsianthuscarbunculus* sp. nov., *Edwardsianthussapphirus* sp. nov., and *Edwardsianthussmaragdus* sp. nov. have two types of basitrichs in their nemathybomes, and *Edwardsianthusamethystus* has no nemathybomes at all. Consequently, we have now added a new character to the diagnosis of this genus: an inequal number of inner and outer tentacles. Species of this genus have a peculiar tentacular arrangement as “5 inner and 15 outer” or “8 inner and 12 outer”. The tentacular arrangement is useful to distinguish *Edwardsianthus* from the genus *Edwardsia* de Quatrefages, 1842 of the same family, as *Edwardsia* species have equal numbers of tentacles in their inner and outer cycles (Carlgren, 1949; Izumi & Fujita, 2019).

#### ﻿ Edwardsianthus pudicus

Taxon classificationAnimaliaActiniariaEdwardsiidae

(Klunzinger, 1877)

11CE36E7-2E17-5D6A-A1F9-58A3E40FC11F

**Figure 2.**
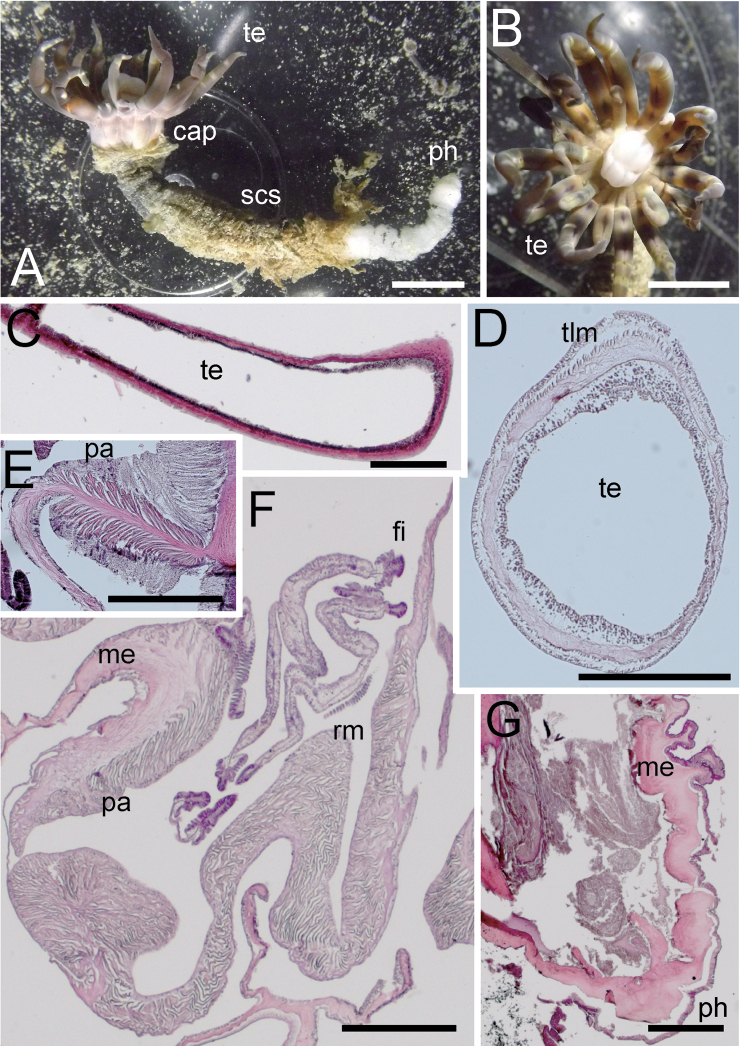
External and internal morphology of *Edwardsianthuspudicus* (NSMT-Co 1702) **A** outer view of living specimen **B** oral view of living specimen **C** longitudinal section of tentacle **D** transverse section of tentacle **E** transverse section of parietal muscle **F** transverse section of macrocnemes **G** longitudinal section of physa. Abbreviations: cap, capitulum; fi, filament; me, mesoglea; pa, parietal muscle; ph, physa; rm, retractor muscle; scs, scapus; te, tentacle; tlm, tentacular longitudinal muscle. Scale bars: 5 mm (**A, B**); 1 mm (**C**); 500 µm (**D, F, G**); 100 µm (**E**).

**Figure 3.**
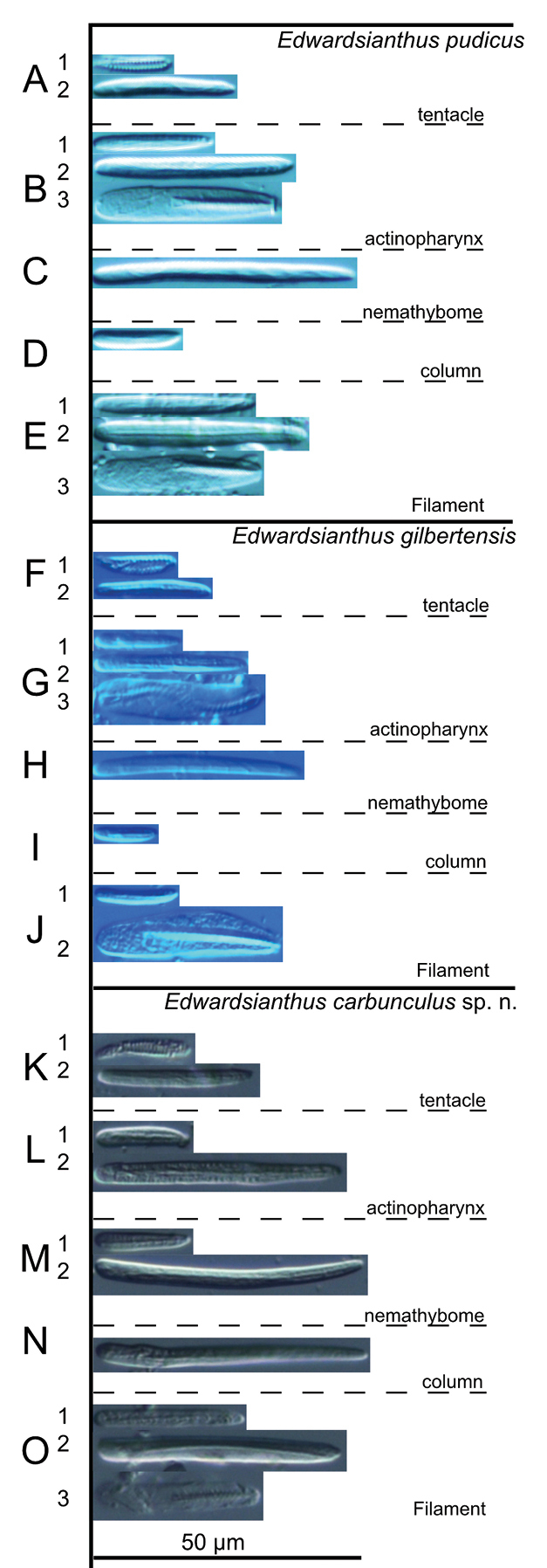
Cnidae of *Edwardsianthus* species **A–E***E.pudicus* (NSMT-Co 1702) **A1** spirocyst in tentacle; **A2** basitrich in tentacle **B1** small basitrich in actinopharynx **B2** large basitrich in actinopharynx **B3** microbasic *p*-mastigophore in actinopharynx **C** basitrich in nemathybome **D** basitrich in column **E1** small basitrich in filament **E2** large basitrich in filament **E3** microbasic *p*- mastigophore in filament **F–J***E.gilbertensis* (CMNH-ZG 06527) **F1** spirocyst in tentacle **F2** basitrich in tentacle **G1** small basitrich in actinopharynx **G2** large basitrich in actinopharynx **G3** microbasic *p*-mastigophore in actinopharynx **H** basitrich in nemathybome **I** basitrich in nemathybome **J1** basitrich in filament **J2** microbasic *p*- mastigophore in filament **K–O***E.carbunculus* sp. nov. (CMNH-ZG 05954) **K1** spirocyst in tentacle **K2** basitrich in tentacle **L1** small basitrich in actinopharynx **L2** large basitrich in actinopharynx **M1** small basitrich in nemathybome **M2** large basitrich in nemathybome **N** basitrich in column **O1** small basitrich in filament **O2** large basitrich in filament **O3** microbasic *p*-mastigophore in filament.

New Japanese name: nanyo-mushimodoki-ginchaku Figs 2 , 3A–E ; Table 4

Edwardsia pudica Klunzinger, 1877: 80–81, pl. 6, fig. 3; Carlgren, 1931: 18–20, figs 16, 17.
Edwardsiella pudica Andres, 1883: 309.
Edwardsia adenensis Faurot, 1895: 121, pl. 6, fig. 5, pl. 7, fig. 6.
Edwardsia bocki Carlgren, 1931: 7–9, figs 5, 6.
Edwardsia stephensoni Carlgren, 1950: 128–129, figs 1, 2.
Edwardsianthus pudica : England, 1987: 224–229, fig. 10.

##### Material examined.

NSMT-Co 1702: histological sections, dissected tissues, and prepared nematocysts, collected by SCUBA diving on 7 November 2015 off Kurasaki seashore, Amami-Oshima Island, Kagoshima, Japan, at ca. 20 m depth, by Takuma Fujii.

##### Description.

***External anatomy*.** Size: ca. 120–200 mm in whole length, and ca. 12–15 mm in width in living specimen, and ca. 80–130 mm in length and ca. 8–10 mm in width in preserved specimen (Fig. 2A). Column: cylinder-like form, and the proximal part narrower to some extent; consisting of capitulum, scapus, and physa. The distal-most part capitulum, translucent and visible magenta mesenteries within, short, without nemathybomes. Scapus with very thick and easily removed periderm-like cuticle, dark gray color in living and preserved animals, and with tiny, pale white in color, densely scattered nemathybomes (Fig. 2A). Tentacles: 20 in number in two cycles, inner tentacle 8 and outer 12, magenta pink or purple in color with brown obscure patches in living animals (Fig. 2B; these colors are lost in preserved specimen), without acrospheres. Inner tentacles short, slender, ca. 5–6 mm in length, and outer ones long and slender, 10–14 mm in length in preserved. Mouth: at the center of oral disc, apparently swollen, showing white color in live specimens. ***Internal anatomy*.** Mesenterial arrangement: eight perfect mesenteries, all macrocnemes. Four dorsal and ventral directives, and four lateral mesenteries not paired with other macrocnemes. All macrocnemes present along whole length of the body from oral to aboral end and bearing distinct retractor and parietal muscles. Twelve tiny microcnemes, without muscles, confined only in distal-most part. Four microcnemes between dorsal directives and dorso-lateral mesenteries, four between dorso-and ventro-lateral mesenteries, and four between ventro-lateral mesenteries and ventral directives. Retractor muscles: at the mid part of column, strongly developed and diffused (Fig. 2F), pennon-like, arranged with ca. 100 muscular processes. Processes except some basal ones simple or slightly branched, and pinnate in some parts. Some processes nearest to body wall extremely well-branched, with secondary and tertiary branches (Fig. 2F; England, 1981: fig. 10). Parietal muscles: developed, comparatively distinct, egg-shaped, elongated along mesenteries, with ca. 15–20 simple or slightly branched muscular processes on each side (Fig. 2E). Others: existence of siphonoglyph unknown because of the contracted state of the specimen. Each with one tentacle from each endo- or exocoels. Tentacular circular muscle indistinct (Fig. 2C), and longitudinal muscle distinct, ectodermal (Fig. 2D). Mesoglea thickest in body wall, > 200 μm in thickness in some parts, and comparatively thick in physa and mesenteries, but thinner in parietal muscles and tentacles (Fig. 2C–E). Nemathybomes sunk into mesoglea. Marginal sphincter muscle and basilar muscle absent (Fig. 2G). Gametogenic tissue not attached to retractor muscles, distinct, but no mature gametocytes observed in our specimen (Fig. 2F). ***Cnidom*.** Basitrichs, spirocysts, and microbasic *p*-mastigophores. See Fig. 3A–E and Table 4 for sizes and distributions of cnidae on this study.

**Table 4.** Cnidoms of the species of *Edwardsianthuspudicus*, *Edwardsianthusgilbertensis* and *Edwardsianthuscarbunculus* sp. nov.

		***Edwardsianthuspudicus***					***Edwardsianthusgilbertensis***					***Edwardsianthuscarbunculus* sp. n.**				
		**NSMT-Co 1702**					**CMNH-ZG 06527**					**CMNH-ZG 05954**				
		Length × Width (µm)	Mean (µm)	SD (µm)	n	frequency	Length × Width (µm)	Mean (µm)	SD (µm)	n	frequency	Length × Width (µm)	Mean (µm)	SD (µm)	n	frequency
Tentacle																
basitrichs		20.1–34.0 × 2.9–4.2	27.4 × 3.6	3.09 × 0.30	57	numerous	13.2–26.4 × 2.8–4.6	20.5 × 3.6	3.23 × 0.44	50	numerous	25.9–34.6 × 2.7–4.3	30.0 × 3.3	2.05 × 0.29	94	numerous
spirocysts		11.9–20.1 × 2.2–3.6	15.1 × 2.9	1.88 × 0.32	25	numerous	8.5–14.3 × 3.0–4.4	11.0 × 3.4	1.43 × 0.35	12	few	11.8–21.0 × 2.5–3.7	16.6 × 3.1	1.83 × 0.28	57	numerous
Actinopharynx																
basitrichs	S	17.4–24.2 × 1.9–3.0	20.8 × 2.5	2.02 × 0.44	15	numerous	16.3–21.1 × 2.4–3.9	18.4 × 3.1	1.24 × 0.39	12	few	14.3–16.9 × 3.5–3.9	16.0 × 3.7	0.99 × 0.19	4	rare
	L	34.8–42.6 × 3.3–5.4	38.4 × 4.5	1.80 × 0.44	47	numerous	26.5–34.3 × 3.0–4.5	30.8 × 3.7	1.71 × 0.35	66	numerous	36.1–48.8 × 3.2–5.0	42.5 × 4.2	2.85 × 0.40	76	numerous
microbasic *p*-mastigophores		30.3–35.6 × 6.6–7.1	33.4 × 6.7	1.92 × 0.17	5	few	28.9–35.5 × 6.6–8.4	32.5 × 7.6	2.71 × 0.78	3	rare	–	–	–	–	–
Nemathybome																
basitrichs	S	–	–	–	–	–	–	–	–	–	–	16.6–19.9 × 3.9–4.1	18.4 × 4.0	1.35 × 0.08	4	rare
	L	46.8–56.6 × 3.2–5.6	41.7 × 51.7	1.95 × 0.43	44	numerou	34.0–45.2 × 3.0–4.8	39.6 × 3.8	2.02 × 0.38	75	numerous	46.8–56.6 × 3.2–5.6	51.7 × 4.3	2.08 × 0.47	44	numerous
Column																
basitrichs		15.8–17.5 × 3.4–3.8	16.8 × 3.7	0.72 × 0.18	3	rare	9.8–14.4 × 2.8–4.1	12.0 × 3.3	1.12 × 0.41	12	few	47.9–53.8 × 3.4–4.8	50.8 × 4.0	1.62 × 0.33	24	numerous
Filament																
basitrichs	S	25.1–31.7 × 2.4–4.1	29.0 × 3.3	1.69 × 0.34	49	numerous	12.9–19.2 × 2.8–4.2	14.8 × 3.4	1.32 × 0.33	61	numerous	22.4–32.2 × 3.2–5.0	28.3 × 4.0	2.29 × 0.43	43	numerous
	L	29.4–42.7 × 4.2–5.9	37.3 × 4.9	3.09 × 0.31	29	numerous	–	–	–	–	–	27.6–44.3 × 4.1–5.8	34.8 × 4.8	5.87 × 0.51	11	few
microbasic *p*-mastigophores		29.7–34.1 × 5.2–7.6	31.6 × 6.2	1.26 × 0.62	11	few	33.0–38.4 × 8.2–10.9	36.3 × 9.3	1.63 × 0.72	12	few	30.1–31.8 × 5.3–5.9	30.9× 5.6	0.87 × 0.29	2	rare

##### Derivation of Japanese name.

see the derivation of genus name.

##### Remarks.

This specimen from Amami Oshima Island resembled the features of *Edwardsianthuspudicus* as stated by England (1987); he redescribed this species as *Edwardsianthuspudica* (Klunzinger, 1877), but the appropriate name is *Edwardsianthuspudicus* following nomenclatural rules (ICZN 31.2 and 34.2; Ride et al., 1999), as in WoRMS (Daly & Fautin, 2021). England (1987) redescribed *E.pudicus* in detail and designated this species as the type of *Edwardsianthus* England, 1987. England (1987) mentioned that *E.pudicus* had a large body, reaching 200 mm in length and 15 mm in width, a thick walled scapus with easily stripped periderm, scattered small nemathybomes, long slender tapered tentacles, swelled mouth, extremely developed and diffused retractor muscles composed of 70–90 muscular processes, well-developed parietal muscle with 20–30 simple or slightly branched processes, and dioecious gametogenic tissue. These features almost completely correspond to the specimen obtained in this study. The tentacles being translucent purple or magenta-pink in color (England, 1987) were also similar to the tentacles and capitulum of our specimen. Moreover, *E.pudicus* inhabits a broad area of the Indo-Pacific region (Fautin, 2013; Daly & Fautin, 2021), so it is not unexpected to find this species in Japanese waters.

#### ﻿ Edwardsianthus gilbertensis

Taxon classificationAnimaliaActiniariaEdwardsiidae

Carlgren, 1931

3736F243-4389-5DFF-BA57-229A0452E87E

Japanese name: minami-mushimodoki-ginchaku: Uchida & Soyama, 2001 Figs 3F–J , 4 ; Table 4

Edwardsia gilbertensis Carlgren, 1931: 10–12, figs 7–9.
Edwardsianthus gilbertensis : England, 1987: 218, 231, fig. 10; Uchida and Soyama, 2001: 49.

##### Material examined.

CMNH-ZG 06527: dissected specimen, histological sections, tissues in paraffin, and prepared nematocysts, collected by wading on 7 June 2013 from the intertidal zone of Kabira Bay, Ishigaki Island, Okinawa Pref., Japan, by Kensuke Yanagi; NSMT-Co 1701: dissected specimens (2 individuals), collected by hand during wading on 26 March 2015 from the intertidal zone of Funaura Bay, Iriomote Island, Okinawa Pref., Japan, by Takato Izumi; NSMT-Co 1781: dissected or whole specimens (3 individuals), collected by wading on 17 March 2015 from the intertidal zone of Yonaha Bay, Miyako Island, Okinawa Pref., Japan, by Takato Izumi; NSMT-Co 1782: histological sections, tissues in paraffin, and prepared nematocysts, collected by wading on 19 March 2014 from the intertidal zone of Kataburu Beach, Yonaguni Island, Okinawa Pref., Japan, by Takato Izumi; NSMT-Co 1783: histological sections, tissues in paraffin, and prepared nematocysts, collected by wading on 23 March 2015 from the intertidal zone of Kataburu Beach, Yonaguni Island, Okinawa Pref., Japan, by Takato Izumi; NSMT-Co 1784–NSMT-Co 1789: whole or dissected specimens, collected by wading on 17 March 2016 from the intertidal zone of Kataburu Beach, Yonaguni Island, Okinawa Pref., Japan, by Takato Izumi; NSMT-Co 1790: dissected specimens (3 individuals), collected by wading on 24 March 2015 from the intertidal zone of Higawa Bay, Yonaguni Island, Okinawa Pref., Japan, by Takato Izumi; NSMT-Co 1791: histological sections, tissues in paraffin, collected by wading on 23 September 2014 from the intertidal zone of Senaga Island, Okinawa Pref., Japan, by Takato Izumi; NSMT-Co 1792: histological sections, tissues in paraffin, collected by wading on 21 September 2014 from the intertidal zone of Yagaji Island, Okinawa Pref., Japan, by Takato Izumi; NSMT-Co 1793: histological sections, tissues in paraffin, collected by hand during snorkeling on 9 November 2015 from Shio-michi, Kikai Island, Kagoshima Pref., Japan, 1 m depth, by Takato Izumi.

##### Description.

**Figure 4.**
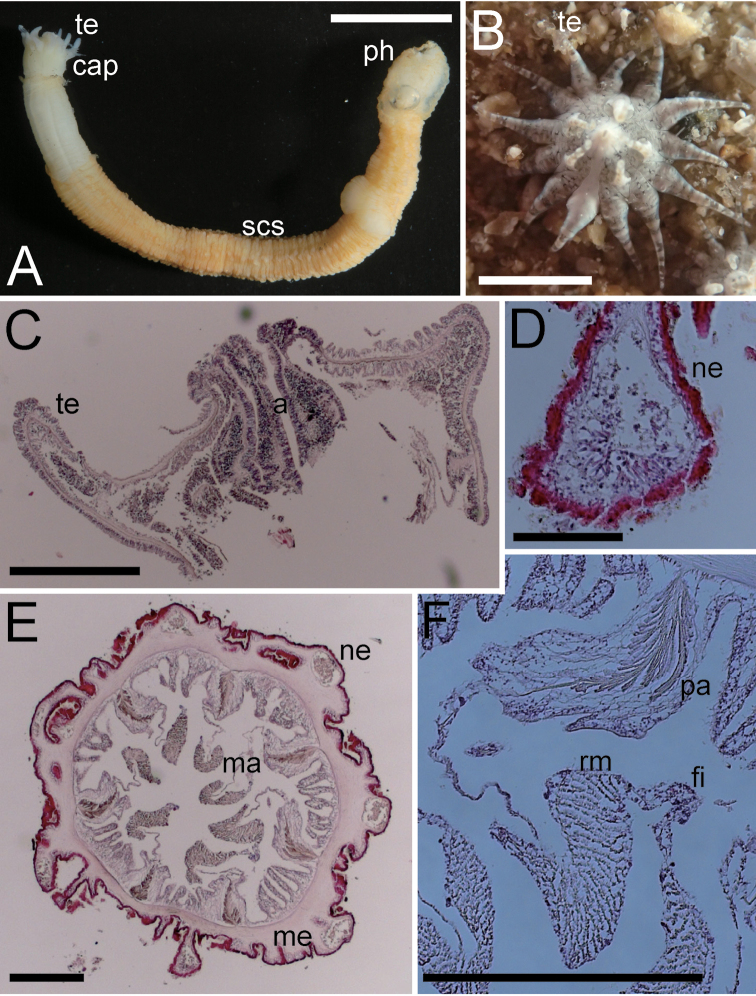
External and internal morphology of *Edwardsianthusgilbertensis* (CMNH-ZG 06527 except for B) **A** outer view of preserved specimen **B** oral view of living specimen with 18 tentacles in the habitat **C** longitudinal section of oral end **D** transverse section of nemathybome **E** transverse section of column in lower part; **F**. Enlarged view of transverse section of mesenteries. Abbreviations: a, actinopharynx; cap, capitulum; fi, filament; ma, macrocneme; ne, nemathybomes; pa, parietal muscle; ph, physa; rm, retractor muscle; scs, scapus; te, tentacle. Scale bars: 5 mm (**A**); 1 mm (**B**); 500 µm (**C, E, F**); 100 µm (**D**). Photograph B by Kensuke Yanagi.

***External anatomy*.** Size: preserved specimens ca. 20–60 mm in whole length, and 2.5–3.5 mm in width, with worm-like form, and equal width along whole body. Column: consisting of capitulum, scapus, and physa. The distal-most part capitulum, translucent or opaque gray in color in living specimens, short, without nemathybomes. Scapus with thick periderm-like cuticle, brownish orange in color, both in living and preserved specimens, and with tiny, pale white in color, more or less in 8 rows nemathybomes. Aboral end apparent physa (Fig. 4A). Tentacles: 20 in number in two cycles; inner tentacles four or five and outer 10–15, opaque whitish gray in color in living animals (Fig. 4B; this color is lost in preserved specimen), without acrospheres. Inner tentacles slender, ca. 1 mm in length, and outer ones long, slender, short, with sparse white spots on surface, 2–3 mm in length. Mouth: at the center of oral disc, a little swollen. ***Internal anatomy*.** Mesenterial arrangement: eight perfect mesenteries, all macrocnemes. Four dorsal and ventral directives, and four lateral mesenteries not paired with other macrocnemes, (Fig. 4E). All macrocnemes are present along the whole body length from oral to aboral end, and bear distinct retractor and parietal muscles. Approximately seven to twelve tiny microcnemes, without muscles, only confined to distal-most part. Four microcnemes between dorsal directives and dorso-lateral mesenteries, four between dorso-and ventro-lateral mesenteries, and four between ventro-lateral mesenteries and ventral directives. Retractor muscles: at the mid part of column, distinct and diffused (Fig. 4E, F), pennon-like, consist of ca. 15–35 simple or slightly branched muscular processes (Fig. 4F). Parietal muscles: distinctly developed, leaf-like shape elongated along mesenteries, with ca. ten simple or slightly branched muscular processes in each side (Fig. 4F). Others: each with one tentacle from each endo- or exocoel. Actinopharynx short, limited in uppermost part (Fig. 4C), without siphonoglyph. Tentacular circular muscle indistinct, and longitudinal muscle distinct, ectodermal. Mesoglea thickest in body wall (Fig. 4E), and comparatively thick in tentacles (Fig. 4C), but thinner in mesenteries and parietal muscles (Fig. 4F). Nemathybomes protruding from mesoglea (Fig. 4D). Marginal sphincter muscle and basilar muscle absent. Gametogenic tissue not attached to retractor muscles, distinct, but no mature gametocyte. Zooxanthellae sparsely distributed on endoderm of mesenteries (Fig. 4F). ***Cnidom*.** Basitrichs, spirocysts, and microbasic *p*-mastigophores. See Fig. 3F–J and Table 4 for sizes and distributions of cnidae.

##### Remarks.

*Edwardsiagilbertensis* was originally described from the Gilbert Islands, Kiribati (Carlgren, 1931), and there have been several reports from other localities in the tropical/subtropical zone in the Pacific (Fautin, 2013). However, there were no records of *E.gilbertensis* from Japan except for a fieldguide (Uchida & Soyama, 2001), which reported this species from Kabira Bay, Ishigaki Island. In this research we discovered many individuals of *E.gilbertensis* living not only in the intertidal zone of Kabira Bay in Ishigaki Island (Fig. 4B), but also across a broad area of the Nansei Islands, from Kikai Island, Amami Islands (Kagoshima Pref.) to Yonaguni Island, Yaeyama Islands (Okinawa Pref.). The morphological features of these specimens almost completely correspond to the original description of Carlgren (1931): 6.5 cm in length and 0.2 cm in width in fixed specimens; 16–20 tentacles; nemathybomes more or less in rows; 20–30 muscular processes on retractors. The cnidom of our specimen also agrees well with the original description, especially concerning the point that there is only one type of cnidae, “31–41 × (2)2.5(3) µ” in size (Carlgren, 1931) in the nemathybomes. Thus, it is confirmed that *Edwardsianthusgilbertensis* is widely distributed in the southern islands of Japan including Ishigaki Island.

#### ﻿ Edwardsianthus carbunculus sp. nov.

Taxon classificationAnimaliaActiniariaEdwardsiidae

EA704A69-959E-541C-9C0F-1550DE68CC12

http://zoobank.org/8AA70F27-89FB-447A-8758-73C0928F8F0E

**Figure 5.**
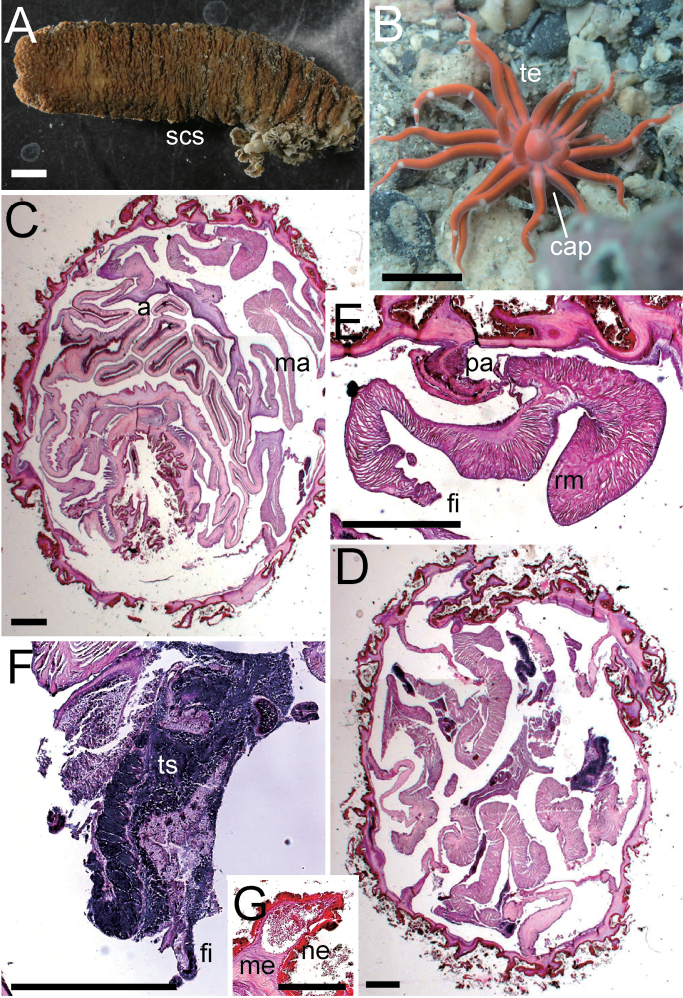
External and internal morphology of *Edwardsianthuscarbunculus* sp. nov. (CMNH-ZG 05954) **A** outer view of preserved specimen (aboral end is damaged) **B** oral view of living specimen in the habitat (photograph Kensuke Yanagi) **C** transverse section of column in upper part **D** transverse section of lower column in lower part **E** transverse section of the macrocneme **F** transverse section of testis **G** transverse section of nemathybomes. Abbreviations: a, actinopharynx; cap, capitulum; fi, filament; me, mesoglea; ne, nemathybomes; pa, parietal muscle; rm, retractor muscle; scs, scapus; te, tentacle. Scale bars: 5 mm (**A, B**); 500 µm (**C–F**); 100 µm (**G**). Picture B taken by Kensuke Yanagi.

Japanese name: rubi-mushimodoki-ginchaku Figs 3K–O , 5 ; Table 4

##### Material examined.

***Holotype***. CMNH-ZG 05954, histological sections, tissues in paraffin, and prepared nematocysts, collected by SCUBA diving on 10 July 2013, Nishidomari (in front of Kuroshio Biological Institute), Kochi Pref., Japan, 5 m depth, by Kensuke Yanagi.

##### Description.

***External anatomy*.** Size: preserved specimen ca. 60 mm in whole length, and 10–15 mm in width (but distal side strongly contracted and aboral side torn off during sampling, so body length estimated > 100 mm when living), with cylinder-like form, and the proximal side a little narrower. Column: consisting of capitulum and scapus. The distal-most part of capitulum, transparent and visible scarlet color inside, short, without nemathybomes. Scapus with very thick periderm-like cuticle, dark brown in color in living specimen (Fig. 5B; this color lost in preserved specimen), and with tiny, pale white in color, densely scattered nemathybomes (Fig. 5A). Tentacles: 20 in number in two cycles: inner tentacles five and outer 15, vivid scarlet in color (Fig. 5B), without acrospheres. Inner tentacles short, blunt, ca. 6 mm in length, and outer ones long, slender, with sparse white spots on surface, 15–20 mm in length in the living specimen. Mouth: at the center of oral disc apparently swollen in living animal (Fig. 5B). ***Internal anatomy*.** Mesenterial arrangement: eight perfect mesenteries, all macrocnemes. Four dorsal and ventral directives, and four lateral mesenteries not paired with other macrocnemes, arranged in normal *Edwardsia* pattern (Fig. 5C, D). All macrocnemes are present along the whole body length from oral to aboral end and bear distinct retractor and parietal muscles. Twelve tiny microcnemes, without muscles, only confined to distal-most part. Four microcnemes between dorsal directives and dorso-lateral mesenteries, four between dorso-and ventro-lateral mesenteries, and four between ventro-lateral mesenteries and ventral directives. Retractor muscles: at the mid part of column, strongly developed and diffused (Fig. 5E), pennon-like, arranged with 60–90 muscular processes, simple or slightly branched, and pinnated in some parts. Some processes nearest to body wall extremely well-branched, into secondary and thirdly branches (Fig. 5E). Parietal muscles: not so well developed, apparently elongated to direction of mesenteries, with ca. 20–30 simple to muscular processes in each side (Fig. 5E). Others: each with one tentacle from each endo- or exocoels. Existence of siphonoglyph unknown because of contracted state of specimen. Tentacular circular muscle indistinct, and longitudinal muscle distinct, ectodermal. Mesoglea thickest in body wall, > 200 μm in thickness in some parts (Fig. 5C, D), and comparatively thick in tentacles and mesenteries (nearby retractor), but thinner in parietal muscles (Fig. 5E). Nemathybomes protruding from mesoglea (Fig. 5G). Marginal sphincter muscle and basilar muscle absent. Gametogenic tissue apart from retractor muscles, distinct, with dense immature testes (Fig. 5F). ***Cnidom*.** Basitrichs, spirocysts, and microbasic *p*-mastigophores. See Fig. 3K–O and Table 4 for sizes and distributions of cnidae.

##### Etymology.

The species epithet refers to ruby, a kind of gemstone, and is named so after the scarlet, vivid red, color of its tentacles. Derivation of the Japanese name is the same as that of the Latin species name.

##### Remarks.

This species can be distinguished from *Edwardsianthus* not only by the scarlet tentacles, the most characteristic feature of this species, and their arrangement, but also by the species’ cnidom: *E.carbunculus* can be distinguished from *E.gilbertensis* and *E.pudicus* by having two types of basitrichs in its nemathybomes (Table 4), and from the other three new species by containing only one type of basitrich in its filaments (Tables 4, 5). In the phylogenetic tree (Fig. 10), *E.carbunculus* sp. nov. is closely related to *E.pudicus*, but there are differences in their morphology as described above and in the separation of their localities: *E.pudicus* inhabits tropical and subtropical waters while *E.carbunculus* were only lives in temperate seas. Therefore, we concluded that this sea anemone is a new species. The genus *Edwardsianthus* is also traditionally characterized by nemathybomes containing only one type of nematocysts, but this definition needs revision because this species has two types of nematocysts in nemathybomes (Fig. 3M, Table 4; compare with the remarks given for the genus *Edwardsianthus*).

**Table 5.** Cnidoms of the species of *Edwardsianthussapphirus* sp. nov., *Edwardsianthussmaragdus* sp. nov., and *Edwardsianthusamethystus* sp. nov.

		***Edwardsianthussapphirus* sp. nov.**					***Edwardsianthussmaragdus* sp. nov.**					***Edwardsianthusamethystus* sp. nov.**				
		CMNH-ZG 09761					CMNH-ZG 09762					CMNH-ZG 09763				
		Length × Width (µm)	Mean (µm)	SD (µm)	n	frequency	Length × Width (µm)	Mean (µm)	SD (µm)	n	frequency	Length × Width (µm)	Mean (µm)	SD (µm)	n	frequency
Tentacle																
basitrichs		27.5–37.3 × 3.0–4.8	33.1 × 3.9	2.27 × 0.40	55	numerous	25.4–40.3 × 3.0–4.7	30.4 × 3.8	2.72 × 0.41	67	numerous	17.0–32.6 × 2.7–4.3	27.7 × 3.6	3.15 × 0.38	55	numerous
spirocysts		14.9–23.6 × 2.8–4.9	19.1 × 3.9	1.78 × 0.46	64	numerous	12.1–21.7 × 2.5–5.1	18.0 × 3.8	2.07 × 0.49	61	numerous	14.6–24.1 × 3.0–4.9	20.6 × 4.0	1.96 × 0.37	53	numerous
Actinopharynx																
basitrichs	S	20.2–25.9 × 2.3–3.6	22.1 × 3.1	1.49 × 0.40	13	few	20.5–23.6 × 2.5–3.0	22.2 × 2.7	1.03 × 0.20	6	few	12.3–18.2 × 2.7–4.7	14.2 × 3.7	1.18 × 0.48	48	numerous
	L	34.1–44.1 × 3.6–5.6	39.1 × 4.7	2.24 × 0.42	44	numerous	34.9–47.7 × 3.5–5.9	40.0 × 4.5	2.46 × 0.44	71	numerous	28.9–49.1 × 3.8–4.7	38.6 × 4.1	9.60 × 0.34	4	rare
microbasic *p*-mastigophores		–	–	–	–	–	35.1–42.0 × 7.2–8.5	38.3 × 7.8	2.73 × 0.49	5	few	–	–	–	–	–
Nemathybome												(No nematocyst was observed)				
basitrichs	S	16.9–20.5 × 3.2–3.9	18.5 × 3.4	1.05 × 0.22	8	few	16.5–17.1 × 3.0–3.7	16.8 × 3.3	0.31 × 0.35	2	rare					
	L	39.8–75.2 × 2.8–4.9	56.0 × 3.7	4.98 × 0.47	43	numerous	48.7–61.6 × 3.0–4.7	54.4 × 3.8	2.75 × 0.41	59	numerous					
Filament																
basitrichs	S	25.2–29.8 × 2.6–3.9	27.1 × 3.4	1.71 × 0.48	4	few	18.6–32.2 × 2.5–4.1	28.2 × 3.1	2.23 × 0.33	61	numerous	12.6–17.3 × 2.9–4.8	15.2 × 3.6	1.15 × 0.45	42	numerous
	L	38.4–50.5 × 4.3–6.2	44.6 × 5.1	2.97 × 0.41	54	numerous	39.3–52.0 × 4.6–7.1	46.1 × 5.8	2.77 × 0.51	45	numerous	27.4–46.3 × 3.6–5.2	36.7 × 4.3	5.98 × 0.41	23	numerous
spirocysts		–	–	–	–	–	15.6–18.2 × 3.0–4.2	16.9 × 3.7	1.31 × 0.57	2	rare	13.3–23.6 × 3.2–6.1	19.5 × 4.8	2.14 × 0.57	27	numerous
microbasic *p*-mastigophores		33.1–42.3 × 5.9–8.3	36.9 × 7.4	2.69 × 0.55	16	numerous	30.9–41.2 × 6.6–8.6	35.0 × 7.8	2.91 × 0.70	13	few	35.1 × 7.8	–	–	1	rare

To complete the description of this species, it is necessary to collect and examine specimens with complete proximal ends. However, this species was collected only once from the type locality, and no additional field observations are known, even despite the presence of its characteristic red tentacles. This species is the only *Edwardsianthus* species inhabiting the temperate zone: the other *Edwardsianthus* species live in tropical or subtropical zones (Fig. 1; Fautin, 2013). Thus, the locality, Kochi, becomes the northernmost distribution limit of this genus.

#### ﻿ Edwardsianthus sapphirus sp. nov.

Taxon classificationAnimaliaActiniariaEdwardsiidae

D4431F22-BDB7-546D-A6FC-73888125A467

http://zoobank.org/84D8919D-0CF0-4C90-BD92-896482C4D206

**Figure 6.**
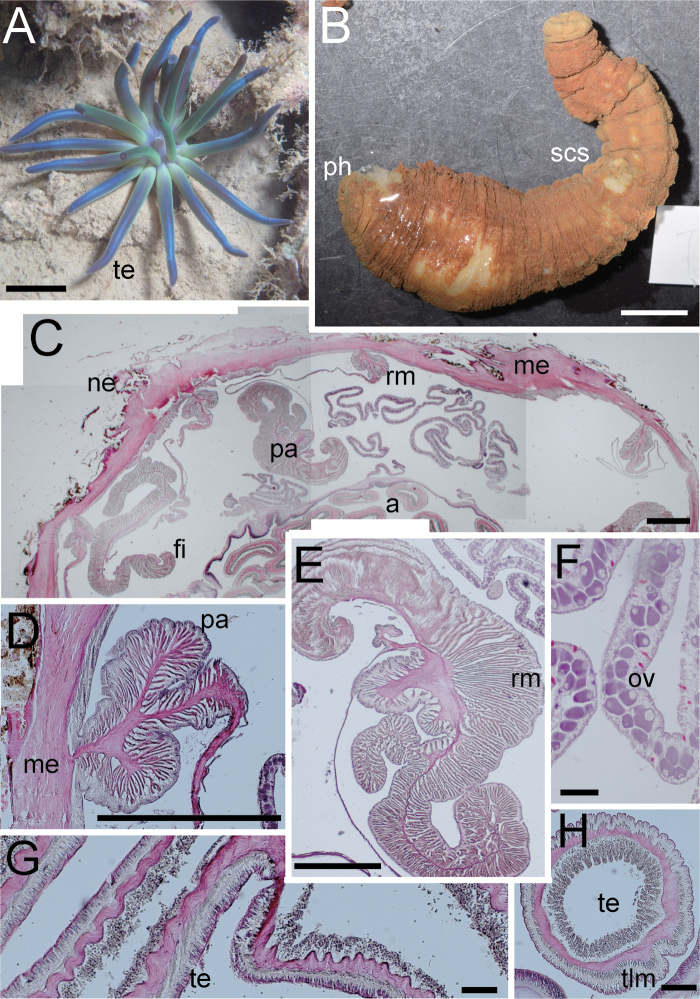
External and internal morphology of *Edwardsianthussapphirus* sp. nov. (CMNH-ZG 09761). **A** oral view of living specimen in the habitat **B** outer view of preserved specimen; **C**. Transverse section of column in upper part **D** enlarged view of transverse section of parietal muscle **E** enlarged view of transverse section of retractor muscle **F** transverse section of ovary **G** longitudinal section of tentacle **H** transverse section of tentacle. Abbreviations: a, actinopharynx; fi, filament; me, mesoglea; ne, nemathybome; oo, oocytes; pa, parietal muscle; ph, physa; rm, retractor muscle; scs, scapus; te, tentacle; tlm, tentacular longitudinal muscle. Scale bars: 1 cm (**A, B**); 500 µm (**C–E**); 100 µm (**F–H**).

**Figure 7.**
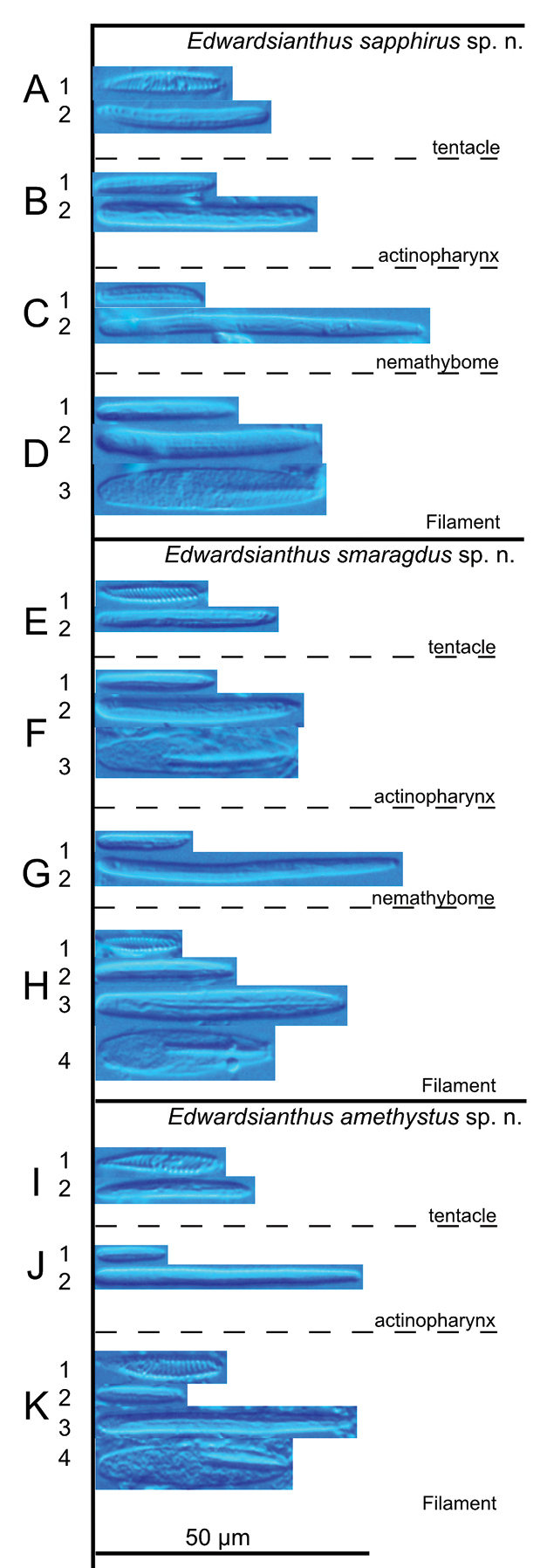
Cnidae of *Edwardsianthus* species **A–D***E.sapphirus* sp. nov. (CMNH-ZG 09761) **A1** spirocyst in tentacle **A2** basitrich in tentacle **B1** small basitrich in actinopharynx **B2** large basitrich in actinopharynx **C1** small basitrich in nemathybome **C2** large basitrich in nemathybome **D1** small basitrich in filament **D2** large basitrich in filament **D3** microbasic *p*-mastigophore in filament **E–H***E.smaragdus* sp. nov. (CMNH-ZG 09762) **E1** spirocyst in tentacle **E2** basitrich in tentacle **F1** small basitrich in actinopharynx **F2** large basitrich in actinopharynx **F3** microbasic *p*-mastigophore in actinopharynx **G1** small basitrich in nemathybome; **G2** large basitrich in nemathybome; **H1** spirocyst in filament; **H2** small basitrich in filament **H3** large basitrich in filament **H4** microbasic *p*-mastigophore in filament **I–K***E.amethystus* sp. nov. (CMNH-ZG 09763) **I1** spirocyst in tentacle **I2** basitrich in tentacle **J1** small basitrich in actinopharynx **J2** large basitrich in actinopharynx **K1** spirocyst in filament **K2** small basitrich in filament **K3** large basitrich in filament **K4** microbasic *p*-mastigophore in filament.

Japanese name: safaia-mushimodoki-ginchaku Figs 6 , 7A–D ; Table 5

##### Material examined.

***Holotype***. CMNH-ZG 09761: histological sections, tissues in paraffin, and prepared nematocysts, collected by SCUBA diving on 24 June 2012, in Oura Bay, Okinawa Island, Okinawa Pref., Japan, 10 m depth, by Takuma Fujii.

##### Description.

***External anatomy*.** Size: preserved specimen ca. 150 mm in whole length, and 20 mm (narrower part)–35 mm (broader part) in width, and > 300 mm in living animal. Column: cylinder-like form, and the proximal part swollen to some extent in preserved specimen. The column consisting of capitulum, scapus and quite small physa. The distal-most part of the capitulum transparently blue, short, without nemathybomes. Scapus with thin and easily stripped periderm, brown in color, and with quite numerous, tiny, pale white in color, scattered nemathybomes (Fig. 6B). Nemathybomes. Aboral end differentiated small, tapered physa. Tentacles: 20 in number in two cycles: inner tentacles 5 and outer 15, metallic greenish blue in color with no pattern in living specimen (Fig. 6A; this color lost in preserved specimen), slender, without acrospheres. Inner tentacles ca. 10 mm and outer ones 15–25 mm in length in the living specimen. Mouth: at the center of oral disc, apparently swollen in living animal (Fig. 6A). ***Internal anatomy*.** Mesenterial arrangement: eight perfect mesenteries, all macrocnemes. Four dorsal and ventral directives, and four lateral mesenteries not paired with other macrocnemes, arranged in normal *Edwardsia* pattern (Fig. 6C). All macrocnemes are present along the whole body length from oral to aboral end and bear distinct retractor and parietal muscles. Twelve tiny microcnemes, without muscles, only confined to distal-most part. Four microcnemes between dorsal directives and dorso-lateral mesenteries, four between dorso-and ventro-lateral mesenteries, and four between ventro-lateral mesenteries and ventral directives. Retractor muscles: at the mid part of column, strongly developed and diffused (Fig. 6E), pennon-like, arranged with 120–150 muscular processes, simple or slightly branched. One process nearest to body wall extremely well-branched, with > 100 secondary and thirdly branched processes (Fig. 6E). Parietal muscles: distinct, developed peculiarly: consisted of ca. 20–30 processes in each side, and only one of them extremely developed, branched into secondary 15–25 processes, and expanded broadly. Thus, parietals in entirety appearing in a characteristic shape like the club symbol of cards (Fig. 6D). Others: each with one tentacle from each endo- or exocoels. Existence of siphonoglyph unknown because of contracted state of specimen. Tentacular circular muscle endodermal, indistinct (Fig. 6G), and longitudinal muscle ectodermal, distinct, and sometimes pinnated (Fig. 6H). Mesoglea thickest in body wall, sometimes reaching 1 mm in thickness (Fig. 6C), but thinner in mesenteries, parietal muscle, and tentacles (Fig. 6E–H). Nemathybomes protruding from mesoglea. Marginal sphincter muscle and basilar muscle absent. Gametogenic tissue apart from retractor muscles, distinct (Fig. 6C, F), with matured oocytes. ***Cnidom*.** Basitrichs, spirocysts, microbasic *p*-mastigophores. See Fig. 7A–D and Table 5 for sizes and distribution.

##### Etymology.

The species epithet refers to a sapphire, a gemstone, and is named so after the brilliant metallic blue color of the species’ tentacles. Derivation of the Japanese name is the same as that of the Latin species name.

##### Remarks.

This species is one of the largest species of its family. It is not only characterized by its gigantic body size, and bluish metallic tentacle coloration, but also by the strange club-like shape of its parietal muscles. Congeneric species have parietal muscles with simple or slightly branched processes, and there are no confirmed cases of parietal muscles with such secondary branched muscular processes in other species. Thus, the shape of parietal muscle of this species is very conspicuous within its genus, allowing *E.sapphirus* to be distinguished easily from its congeners.

There have been several observations of the metallic blue tentacles resembling this species reported during SCUBA diving in Amami Oshima by Takuma Fujii and some other divers (Atetsu Bay and some other places). However, it was too difficult to dig out such large edwardsiid sea anemones that are buried deeply in the substrate, as they usually retract their whole bodies quickly into the substrate. Therefore, we think that the difficulty in collecting multiple specimens is the most serious issue that needs to be overcome in order to make additional progress in the study of edwardsiids.

#### ﻿ Edwardsianthus smaragdus sp. nov.

Taxon classificationAnimaliaActiniariaEdwardsiidae

E7110EE8-8029-5AC7-9259-2D11B062ADC0

http://zoobank.org/05B8173F-21FB-4429-B5BB-5371E3EB144F

**Figure 8.**
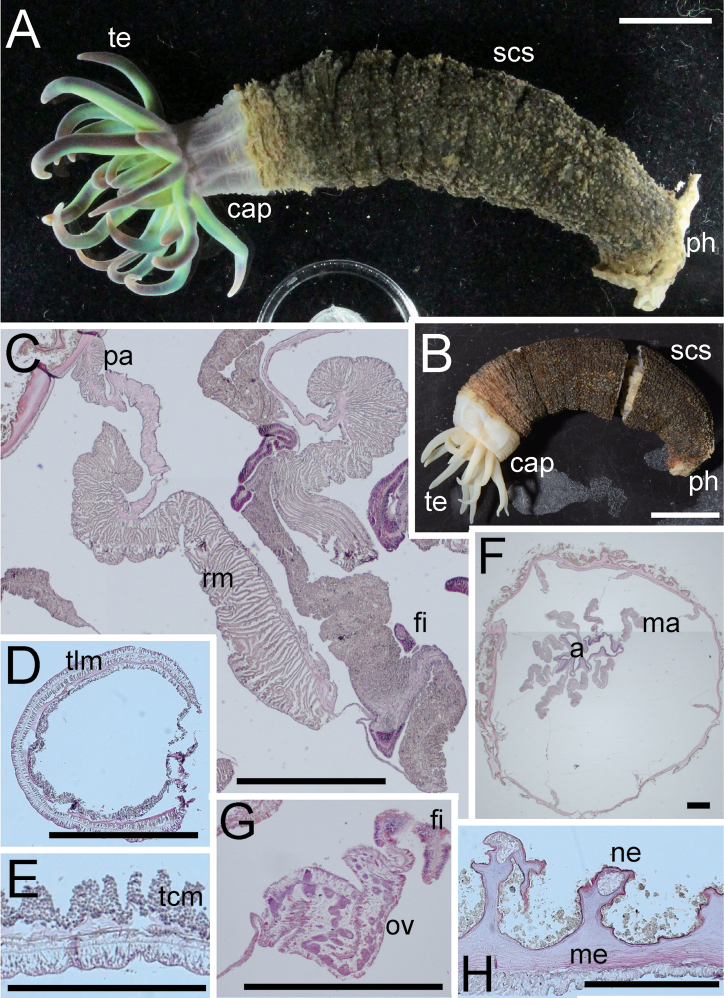
External and internal morphology of *Edwardsianthussmaragdus* sp. nov. (CMNH-ZG 09762). **A** outer view of living specimen **B** outer view of preserved specimen **C** transverse section of retractor muscle **D** transverse section of the tentacle **E** longitudinal section of the tentacle **F** transverse section of column **G** enlarged view of transverse section of ovary **H** transverse section of a nemathybome. Abbreviations: a, actinopharynx; cap, capitulum; fi, filament; ma, macrocneme; me, mesoglea; ne, nemathybomes; ov, ovary; pa, parietal muscle; ph, physa; rm, retractor muscle; scs, scapus; te, tentacle; tcm, tentacular circular muscle; tlm, tentacular longitudinal muscle. Scale bars: 1 cm (**A, B**); 500 µm in (**C**–**H**).

Japanese name: emerarudo-mushimodoki-emeginchaku Figs 7E–H , 8 ; Table 5

##### Material examined.

***Holotype***. CMNH-ZG 09762: histological sections, tissues in paraffin, and prepared nematocysts, collected by SCUBA diving on 31 January 2016, off Shirahama seashore, Amami-Oshima Island, Kagoshima, Japan, 15 m depth, by Daisuke Uyeno.

##### Description.

***External anatomy*.** Size: preserved specimen ca. 70 mm in whole length, and ca. 15 mm in width, and ca. 100 mm in living specimen. Column: cylinder-like in form, and the middle part swollen to some extent (Fig. 8A, B). The column consisting of capitulum, scapus and quite small physa. The distal-most part of the capitulum whitish transparent in living animals, short, without nemathybomes. Scapus with thick periderm, brownish black in color, and with protruding scattered tiny, dingy grey color nemathybomes in the living specimen (Fig. 8A). Aboral end differentiated small, tapered physa. Tentacles: 20 in number in two cycles: inner tentacles five and outer 15, brilliant green in color and pale purple at the tips, no pattern, comparatively slender, without acrospheres. Inner tentacles ca. 7 mm and outer ones ca. 10–15 mm in length in the living specimen. Mouth: at the center of oral disc, slightly swollen both in living and preserved specimen. ***Internal anatomy*.** Mesenterial arrangement: eight perfect mesenteries, all macrocnemes. Four dorsal and ventral directives, and four lateral mesenteries not paired with other macrocnemes, arranged in normal *Edwardsia* pattern (Fig. 8F). All macrocnemes are present along the whole body length from oral to aboral end and bear distinct retractor and parietal muscles. Twelve tiny microcnemes, without muscles, only confined to distal-most part. Four microcnemes between dorsal directives and dorso-lateral mesenteries, four between dorso-and ventro-lateral mesenteries, and four between ventro-lateral mesenteries and ventral directives. Each tentacle exo- or endocoelic. Retractor muscles: at the mid part of column, weakly developed but distinct, diffused (Fig. 8C), pennon-like, arranged with 50–60 muscular processes, simple or slightly branched. One process nearest to body wall well-branched (Fig. 8C). Parietal muscles indistinct, elongated in direction of mesenteries, consisted of short and slightly branched processes, sparsely, < ten on each side (Fig. 8C). Others: each with one tentacle from each endo- or exocoels. Existence of siphonoglyph unknown because of contracted state of specimen. Tentacular circular muscle endodermal, distinct, and longitudinal muscle ectodermal, both distinct. Mesoglea thickest in body wall and actinopharynx, maximum 400 µm in thickness (Fig. 8F), but far thinner in parietal muscle and tentacles (Fig. 8C–E), and thinnest, < 10 µm, in mesenteries. Nemathybomes protruding from mesoglea (Fig. 8H). Marginal sphincter muscle and basilar muscle absent. Gametogenic tissue apart from retractor muscles, distinct (Fig. 8G), with matured oocytes. ***Cnidom*.** Basitrichs, spirocysts, microbasic *p*-mastigophores. See Fig. 7E–H and Table 5 for sizes and distribution.

##### Etymology.

This species epithet refers to an emerald, a gemstone, and is named so after the bright green coloration of its tentacles. Derivation of the Japanese name is the same as that of the Latin species name.

##### Remarks.

**Figure 9.**
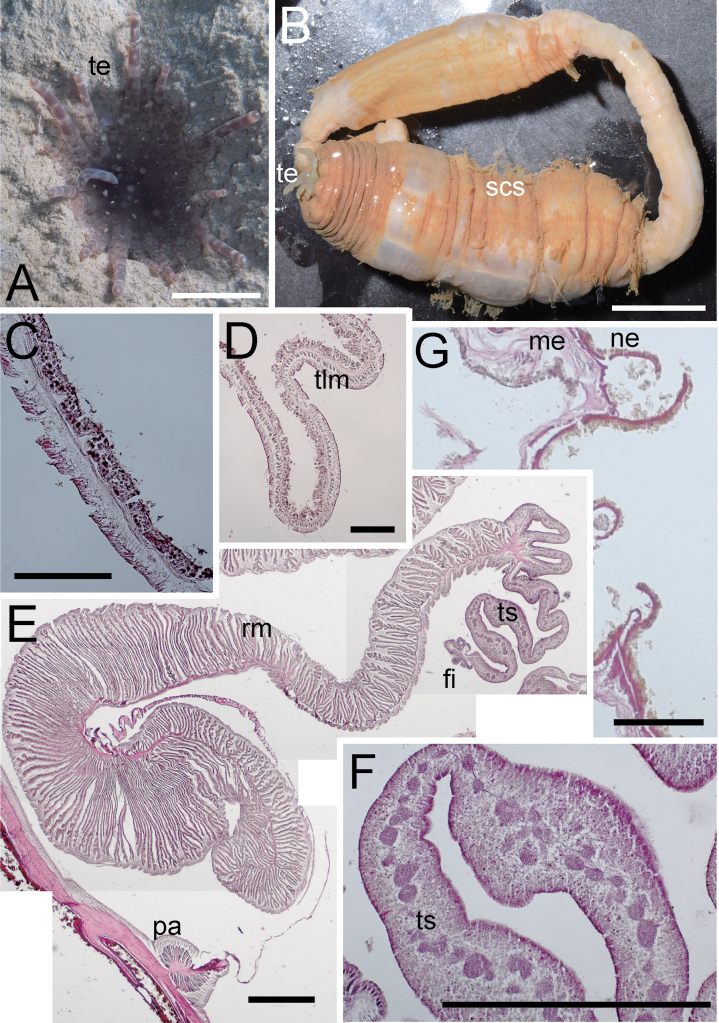
External and internal morphology of *Edwardsianthusamethystus* sp. nov. (CMNH-ZG 09763) **A** oral view of living specimen in the habitat **B** outer view of preserved specimen **C** longitudinal section of tentacle **D** transverse section of tentacle **E** transverse section of retractor muscle **F** transverse section of testis **G** transverse section of trace of nemathybome. Abbreviations: fi, filament; me, mesoglea; ne, nemathybome-like structure; pa, parietal muscle; rm, retractor muscle; scs, scapus; te, tentacle; tlm, tentacle longitudinal muscle; ts, testis. Scale bars: 1 cm (**A, B**); 500 µm (**E, F**); 100 µm (**C, D, G**).

*Edwardsianthus* species usually have strongly developed and diffused retractor and parietal muscles (Figs 2F, 4F, 5E, 6E, 9E), but those of *Edwardsianthussmaragdus* form an exception by their less distinct development (Fig. 8F). This character is clear in addition to its brilliant light green tentacles. Concerning the cnidom, *E.smaragdus* can be distinguished from *E.pudicus* and *E.gilbertensis* by containing two types of basitrichs in its nemathybomes, and from the other three new species of *Edwardsianthus* by having microbasic *p*-mastigophores in its actinopharynx (Tables 4, 5).

**Figure 10.**
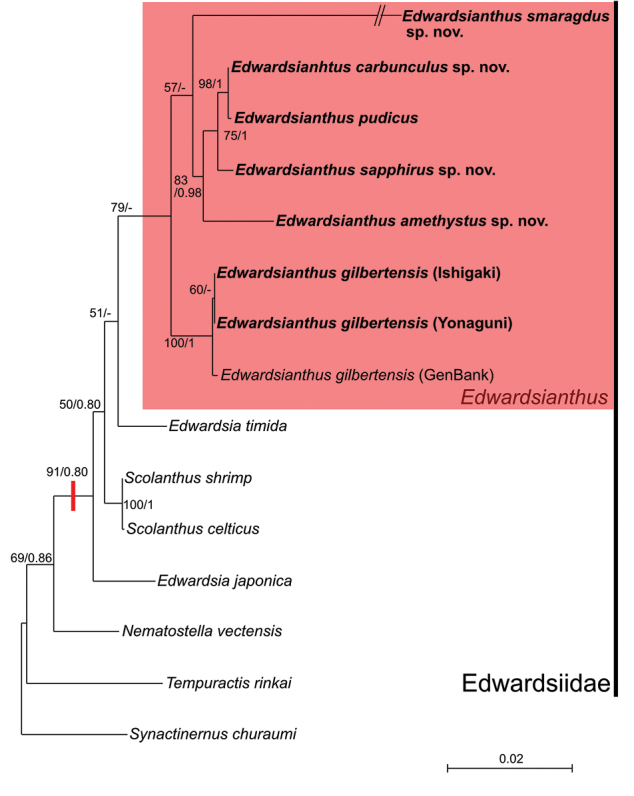
Maximum-likelihood tree of the order Actiniaria based on the combined dataset of mitochondrial 12S and 16S and nuclear 18S rDNA (total 2866 bp). The clade of the genus *Edwardsianthus* is colored in a red box. Red bar at node indicates the position at which nemathybomes would be obtained. Numbers indicate ML bootstrap support values followed by BI posterior probabilities of the nodes (bootstrap values of ≥ 50% and posterior probabilities ≥ 0.5 are shown).

In the phylogenetic tree (Fig. 10; Suppl. material 1 Fig. S1), *E.smaragdus* sp. nov. has a far longer branch than the other species, and therefore its phylogenetic position is not stable; the ML bootstrap value was 57, which is comparatively low, and not supported by BI posterior probability; Fig. 10). Nevertheless, it is most probable that *E.smaragdus* n. sp. belongs to this genus (ML bootstrap value was 79) despite the BI posterior probability not being well-supported. Considering that the morphology of this species corresponds completely with the diagnosis of *Edwardsianthus*, this species is classified as *E.smaragdus*.

#### ﻿ Edwardsianthus amethystus sp. nov.

Taxon classificationAnimaliaActiniariaEdwardsiidae

2A35ED1E-0664-500F-8461-A6EFFFC6C926

http://zoobank.org/E472474A-8E8B-4B30-93CC-B46005F3F8F0

Japanese name: amejisuto-mushimodoki-ginchaku Figs 7I–K , 9 ; Table 5

##### Material examined.

***Holotype***. CMNH-ZG 09763: histological sections, tissues in paraffin, and prepared nematocysts, collected by SCUBA diving on 28 March 2013, in Oura Bay, Okinawa Island, Okinawa Pref., Japan, 15 m depth, by Takuma Fujii.

##### Description.

***External anatomy*.** Size: preserved specimen ca. 200 mm in whole length, and 7 mm (narrower part)–20 mm (broader part) in width, and > 300 mm in living animal, one of the largest species in edwardsiids (Fig. 9B). Column: worm-like in form, and the distal part swollen to some extent (maybe because of condition during preservation). The column consisting of capitulum, scapus and quite small physa. The distal-most part a short capitulum, without nemathybomes. Scapus with thin and easily stripped periderm, light brown in color, and surface completely smooth, with extremely small nemathybome-like spots (Fig. 9B). Aboral end differentiated with small, rounded physa. Tentacles: 20 in number in two cycles: inner tentacles five and outer 15, slender, pale purple in color with several dark purple spots (Fig. 9A; this color is lost in preserved specimen: Fig. 9B). Inner tentacles ca. 10 mm and outer ones ca. 15–20 mm in length in the living specimen. Mouth: at the center of oral disc, apparently swollen in living animal. ***Internal anatomy*.** Mesenterial arrangement: eight perfect mesenteries, all macrocnemes. Four dorsal and ventral directives, and four lateral mesenteries not paired with other macrocnemes, arranged in the normal *Edwardsia* pattern. All macrocnemes are present along the whole body length from oral to aboral end and bear distinct retractor and parietal muscles. Twelve tiny microcnemes, without muscles, only confined to distal-most part. Four microcnemes between dorsal directives and dorso-lateral mesenteries, four between dorso-and ventro-lateral mesenteries, and four between ventro-lateral mesenteries and ventral directives. Retractor muscles: at the mid part of column, strongly developed and diffused (Fig. 9E), pennon-like, arranged with 100–150 muscular processes, simple to well branched. Processes near filament short and highly branched, and one process nearest to the body wall extremely well-branched, with ca. 80 secondary and thirdly branched processes (Fig. 9E). Parietal muscles: distinct, rounded shape, consisting of 10–15 simple processes on each side (Fig. 9E). Others: each with one tentacle from each endo- or exocoels. Existence of siphonoglyph unknown because of contracted state of specimen. Tentacular circular muscle indistinct (Fig. 9C), and longitudinal muscle ectodermal, distinct (Fig. 9D). Mesoglea thickest in body wall and actinopharynx, ca. 200 µm in thickness (Fig. 9E), but far thinner in mesenteries, retractor muscles, and tentacles (Fig. 9C, D). Nemathybome-like structures protruding from mesoglea, but without any nematocysts (Fig. 9G). Marginal sphincter muscle and basilar muscle absent. Gametogenic tissue apart from retractor muscles, distinct (Fig. 9F), with matured oocytes. ***Cnidom*.** Basitrichs, spirocysts, and microbasic *p*-mastigophores. There are no nematocysts in nemathybome-like structures. See Fig. 7I–K and Table 5 for sizes and distributions.

##### Etymology.

This species epithet refers to amethyst, a kind of gemstone, and is named after this species’ dark purple tentacle coloration. Derivation of the Japanese name is the same as that of the Latin species name.

##### Remarks.

The most characteristic feature of this species is the nemathybome-like features without nematocysts. Nemathybomes are pocket-like features on columns of some genera of Edwardsiidae, and they always contain large nematocysts (Carlgren, 1949; Brandão et al. 2019). Thus, the structures of *Edwardsianthusamethystus* cannot be called nemathybomes because they lack nematocysts. This is the first case of confirmation of this nemathybome-like feature in *Edwardsianthus* anemones, and by these *E.amethystus* can be easily distinguished from its congeners. We placed this sea anemone in the genus *Edwardsianthus* because of the characteristic arrangement of tentacles and mesenteries, but the generic diagnosis has now been modified to “sometimes without” nemathybomes (see the Remarks for the genus).

##### Phylogenetic analyses.

The concatenated phylogenetic tree of 12S, 16S, and 18S rDNA (total 2886 bp) is shown in Fig. 10. All *Edwardsianthus* specimens formed a clade (indicated in red box) supported by a ML bootstrap value of 79%, but not well supported by BI posterior probability. In this clade, *E.pudicus*, *E.carbunculus* sp. nov., *E.sapphirus* sp. nov., and *E.amethystus* sp. nov. were closely related with high support (ML bootstrap value = 83%; BI posterior probability = 0.98), *Edwardsianthussmaragdus* sp. nov. was indicated as their sister group, but was only slightly supported by ML (bootstrap value = 57%) and not supported by the BI method. *Edwardsianthusgilbertensis* was nested with the other five species and positioned at the most basal node of this genus.

In addition, the most basal position of our phylogenetic tree of Edwardsiidae is taken by *Tempuractisrinkai* Izumi, Ise, & Yanagi, 2018. This edwardsiid is the only species of the genus *Tempuractis* Izumi, Ise, & Yanagi, 2018. It has a simple morphology compared to other edwardsiid species by showing a smooth body wall without particular structures, like nemathybomes, a simple aboral end without any apparent physa, and simple tentacles without any structures (Izumi et al., 2018). This topology suggests that nemathybomes of Edwardsiidae were obtained within the family lineage (Fig. 10).

## Supplementary Material

XML Treatment for Edwardsianthus

XML Treatment for Edwardsianthus pudicus

XML Treatment for Edwardsianthus gilbertensis

XML Treatment for Edwardsianthus carbunculus

XML Treatment for Edwardsianthus sapphirus

XML Treatment for Edwardsianthus smaragdus

XML Treatment for Edwardsianthus amethystus

## Supplementary materials

Supplementary material 1 The correct shape of Maximum-likelihood tree of the order Actiniaria based on the combined dataset of mitochondrial 12S and 16S and nuclear 18S rDNA (total 2866 bp) Data type phylogenetic tree https://binary.pensoft.net/file/621146This dataset is made available under the Open Database License (http://opendatacommons.org/licenses/odbl/1.0/). The Open Database License (ODbL) is a license agreement intended to allow users to freely share, modify, and use this Dataset while maintaining this same freedom for others, provided that the original source and author(s) are credited.Takato Izumi, Takuma Fujii
