# Supplementary figures and images for: ﻿Gems of the southern Japanese seas – four new species of Edwardsianthus (Anthozoa, Actiniaria, Edwardsiidae) with redescriptions of two species
Source: Zookeys. 2021 Dec 10;1076:151–82. doi: 10.3897/zookeys.1076.69025 (PMC8683394; doi:10.3897/zookeys.1076.69025)

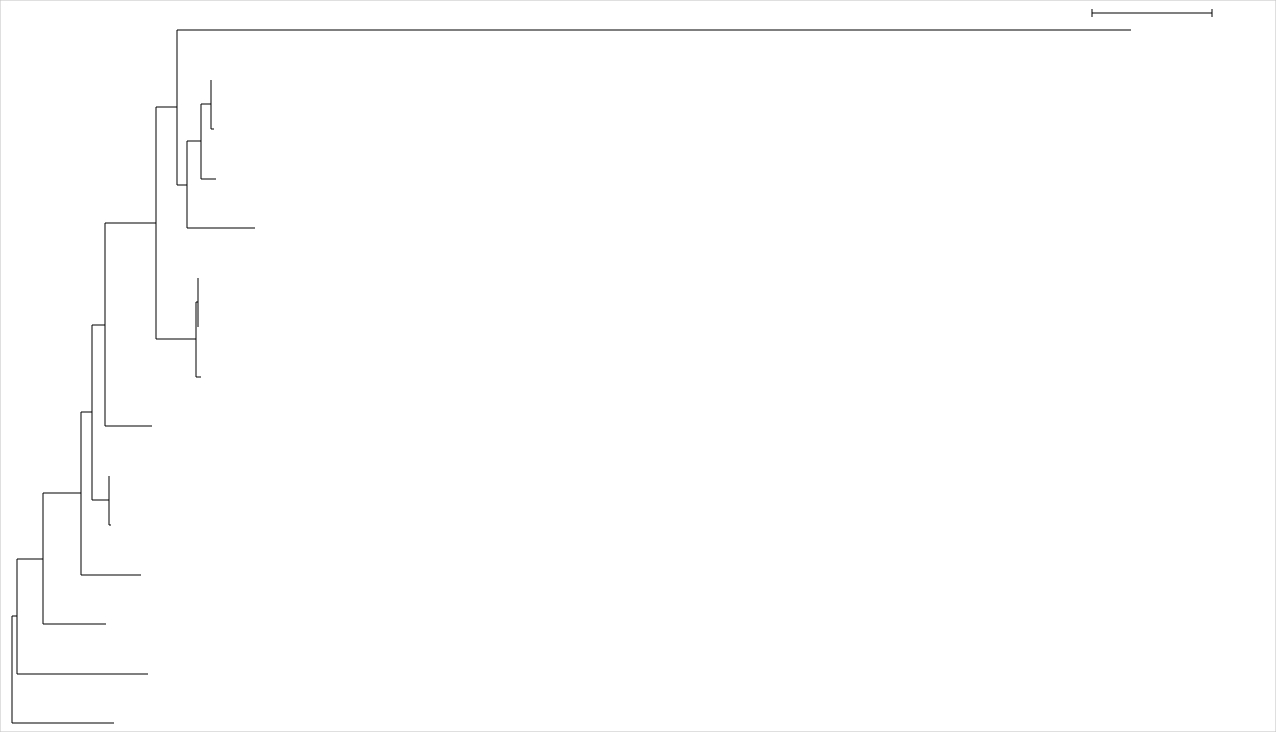

Supplement: Supplementary material 1 — The correct shape of Maximum-likelihood tree of the order Actiniaria based on the combined dataset of mitochondrial 12S and 16S and nuclear 18S rDNA (total 2866 bp) [file zookeys-1076-151-s001.crdownload]
